# *Bsep/Abcb11* knockout ameliorates *Schistosoma mansoni* liver pathology by reducing parasite fecundity

**DOI:** 10.1111/liv.15710

**Published:** 2023-08-29

**Authors:** Tomáš Macháček, Claudia D. Fuchs, Franziska Winkelmann, Marcus Frank, Hubert Scharnagl, Tatjana Stojakovic, Martina Sombetzki, Michael Trauner

**Affiliations:** ^1^ Division of Tropical Medicine and Infectious Diseases Center of Internal Medicine II Rostock University Medical Center Rostock Germany; ^2^ Department of Parasitology Faculty of Science Charles University Prague Czechia; ^3^ Hans Popper Laboratory of Molecular Hepatology Division of Gastroenterology and Hepatology Department of Internal Medicine III Medical University of Vienna Vienna Austria; ^4^ Medical Biology and Electron Microscopy Center University Medical Center Rostock Rostock Germany; ^5^ Department Life Light and Matter University of Rostock Rostock Germany; ^6^ Clinical Institute of Medical and Chemical Laboratory Diagnostics Medical University of Graz Graz Austria; ^7^ Clinical Institute of Medical and Chemical Laboratory Diagnostics University Hospital Graz Graz Austria

**Keywords:** anti‐inflammatory, bile acids, infection, liver fibrosis, *Schistosoma mansoni*

## Abstract

**Background and Aims:**

*Schistosoma mansoni* infection is one of the worldwide leading causes of liver fibrosis and portal hypertension. The objective of this study was to evaluate whether polyhydroxylated bile acids (BAs), known to protect mice from the development of acquired cholestatic liver injury, counteract *S. mansoni*‐induced inflammation and fibrosis.

**Methods:**

Adult FVB/N wild type (WT) and *Abcb11*/*Bsep* ^−/−^ mice were infected with either 25 or 50 *S. mansoni* cercariae. Eight weeks post infection, effects on liver histology, serum biochemistry, gene expression profile of proinflammatory cytokines and fibrotic markers, hepatic hydroxyproline content and FACS analysis were performed.

**Results:**

*Bsep* ^−/−^ mice infected with *S. mansoni* showed significantly less hepatic inflammation and tendentially less fibrosis compared to infected WT mice. Despite elevated alanine aminotransferase, aspartate aminotransferase and alkaline phosphatase levels in infected *Bsep* ^−/−^ mice, inflammatory cells such as M2 macrophages and Mac‐2/galectin‐3^+^ cells were reduced in these animals. Accordingly, mRNA‐expression levels of anti‐inflammatory cytokines (IL‐4 and IL‐13) were increased in *Bsep* ^−/−^ mice upon infection. Furthermore, infected *Bsep^−/−^* mice exhibited decreased hepatic egg load and parasite fecundity, consequently affecting the worm reproduction rate. This outcome could arise from elevated serum BA levels and lower blood pH in *Bsep* ^−/−^ mice.

**Conclusions:**

The loss of *Bsep* and the resulting changes in bile acid composition and blood pH are associated with the reduction of parasite fecundity, thus attenuating the development of *S. mansoni*‐induced hepatic inflammation and fibrosis.

**Abbreviations**
*Acta2*
: actin alpha 2
ALT: alanine aminotransferase
AP: alkaline phosphatase
AST: aspartate aminotransferase
BA: bile acid
BSEP: bile salt export pump
CD: cluster of differentiation
*Col1a2*
: collagen 1a2
CTLA4: cytotoxic T‐lymphocyte‐associated protein 4
FCS: foetal calf serum
FOXP3: Forkhead‐box‐protein P3
*Ifng*
: interferon gamma
IL: interleukin
Mac‐2/galectin3: galactose‐specific lectin 3
*Mdr2*
: multidrug resistance protein 2
norUDCA/NCA: nor‐ursodeoxycholic acid/norucholic acid
PBS: phosphate‐buffered saline
PSR: picrosirius red
qRT‐PCR: quantitative real‐time polymerase chain reaction
*Tgfb*
: transforming growth factor beta
*Tnfa*
: tumour necrosis factor alpha
UDCA: ursodeoxycholic acid
wpi: weeks post infection
WT: wild type

**Key Points**

Hepatic schistosomiasis is a worldwide leading cause of liver fibrosis and portal hypertension. In mice lacking the major hepatic excretory system for bile acids (BAs), bile salt export pump (*Bsep/Abcb11*), accumulation of hydrophilic BAs mitigates hepatic schistosomiasis (liver fibrosis and inflammation). The protective effect is mediated by reduction of the parasite ability to produce eggs, which are responsible for the tissue pathology.

## 1 INTRODUCTION

Development of hepatic fibrosis is the result of an imbalance between production and dissolution of extracellular matrix. One of the worldwide leading causes for hepatic fibrosis is *Schistosoma mansoni* infection,^1^, ^2^ which subsequently leads to portal hypertension and variceal bleeding.^1^, ^3^ *Schistosoma mansoni*‐related hepatic fibrosis is mainly induced via the immune response mediated by CD4^+^ T helper (Th) cells. While the Th1 response dominates in the first weeks of *S. mansoni* infection (when immature parasite stages migrate in the host), the switch to Th2 polarization occurs after 6–8 weeks. It is triggered by strong immunomodulatory antigens released from eggs laid by adult parasites dwelling in the mesenteric veins.^4^, ^5^ Large amounts of eggs are entrapped in the liver, where they induce the formation of periovular granulomas composed mostly of eosinophils, macrophages and lymphocytes.^2^ Such granulomatous reaction is orchestrated by Th2 cytokines that also activate hepatic stellate cells, leading to massive collagen production and liver fibrosis with severe consequences (e.g. portal hypertension, ascites and oesophageal varices).^2^, ^6^, ^7^, ^8^ Despite effective anthelminthic therapy, in many cases *S. mansoni* infection‐related portal hypertension and its complications may persist, thus indicating the urgent need for novel treatment strategies for *S. mansoni*‐induced liver fibrosis.^9^, ^10^ The hydrophilic side chain‐shortened bile acid (BA) 24‐nor‐ursodeoxycholic acid (norUDCA), showing promising results in phase 2 studies for fatty liver disease as well as for primary sclerosing cholangitis,^11^, ^12^ was effective in counteracting *S. mansoni*‐induced hepatic fibrosis due to its immunomodulatory effects resulting in reduced T‐lymphocyte proliferation.^13^ In addition, another hydrophilic BA species, namely tetrahydroxylated BAs (THBAs; the most prominent BA species in mice lacking the hepatic bile salt export pump *Abcb11/Bsep*), were shown to protect mice from the development of acute or chronic cholestatic liver injury induced either by bile duct ligation or 3,5‐diethoxycarbonyl‐1,4‐dihydrocollidine feeding.^14^ Furthermore, THBAs also prevented development of liver inflammation and fibrosis in the *Mdr2* ^−/−^ mouse model of sclerosing cholangitis.^15^

Therefore, the aim of the current study was to investigate the potential therapeutic relevance of THBAs in counteracting *S. mansoni*‐induced liver injury using the Bsep/Abcb11 knockout (*Bsep* ^−/−^) mice.

## 2 MATERIALS AND METHODS

### 2.1 Animals and *Schistosoma mansoni* infection

*Bsep* ^−/−^ and wild type (WT) FVB/N mice were kindly provided by the British Columbia Cancer Research Center.^16^ Age‐matched females (14–15 weeks old) were percutaneously infected with a defined number of *S. mansoni* (Belo Horizonte strain) cercariae freshly emerged from freshwater snails *Biomphalaria glabrata* (Brazilian strain). The parasite life cycle is routinely maintained at the University Medical Center, Rostock. The infection was performed according to the well‐established “water bath” protocol.^17^, ^18^

To assess the outcome of *S. mansoni* infection, the mice were infected with 25 or 50 cercariae (25: *n*_WT_ = 8, $n_{{\mathrm{Bsep}}^{‐/‐}}$ = 7; 50: *n*_WT_ = 8, $n_{{\mathrm{Bsep}}^{‐/‐}}$ = 9) and harvested 8 weeks postinfection (wpi). Uninfected mice (*n*_WT_ = 8, $n_{{\mathrm{Bsep}}^{‐/‐}}$ = 4) were used as naive controls. Blood, liver, spleen and distal colon were collected and further processed. To obtain parasites for phenotype analysis and in vitro experiments, the mice (*n*_WT_ = 9, $n_{{\mathrm{Bsep}}^{‐/‐}}$ = 9) were infected with 300 cercariae and harvested 6 wpi. Mice were always sacrificed via cervical dislocation under deep ketamine/xylazine anaesthesia. All efforts were made to minimize animal suffering.

### 2.2 Serum biochemistry

Blood was collected at harvesting and centrifuged for 20 min at 1500 *g*. Serum was stored at −80°C until analysis. Levels of transaminases (aspartate aminotransferase, AST; alanine aminotransferase, ALT), alkaline phosphatase (AP; Roche Diagnostics) and BAs (DiaSys Diagnostic Systems GmbH) were measured using enzymatic methods according to the manufacturer's instructions.

### 2.3 Parasite‐specific serum IgG

Levels of parasite‐specific serum IgG were measured by in‐house ELISA. Briefly, the microplate wells were coated with soluble worm antigen (1 μg/mL), blocked with 1% bovine serum albumin in phosphate‐buffered saline (PBS)‐0.05% Tween‐20 and probed with the serum (1:100). Anti‐mouse IgG peroxidase‐conjugated antibody (1:8000; Merck, #A2554) was added, and TMB liquid substrate (Merck, #T0440) was finally used for visualization of the reaction.

### 2.4 Liver histology and immunohistochemistry

Standardized liver lobes were fixed in 4% neutral‐buffered formaldehyde solution and embedded in paraffin. Four‐μm‐thick sections were stained with haematoxylin and eosin or picrosirius red (PSR) to assess histopathology or fibrosis, respectively.^19^

Alternatively, immunohistochemical detection of the Mac2/galectin‐3 (the macrophage marker) was performed to characterize the hepatic inflammation. Briefly, the sections were deparaffinated, rehydrated and digested with 0.1% protease. Endogenous peroxidase was blocked with 1% H_2_O_2_ in methanol. Specific binding of the anti‐Mac‐2 monoclonal antibody (clone M3/38; Cedarlane, #CL8942AP) was detected using a biotinylated anti‐rat IgG and the ABC‐System with amino‐9‐ethyl‐carbazole as substrate.

For quantification, whole tissue sections were scanned using slide scanner VS200 (Olympus) and analysed with the HALO image analysis platform (Indica Labs). Representative parts of the whole scans are shown in the manuscript. Files of whole liver sections are available upon request.

### 2.5 Hepatic hydroxyproline content

To quantify liver fibrosis, hepatic hydroxyproline was measured from a standardized liver lobe by Total Collagen Assay Kit (QuickZyme Biosciences), which is based on the colorimetric determination of hydroxyproline residues obtained by acid hydrolysis of collagen.

### 2.6 Quantitative real‐time polymerase chain reaction analysis of gene expression

Total RNA was isolated (RNeasy Plus Mini Kit, Qiagen) from snap‐frozen standardized liver lobes and reversely transcribed into cDNA (High‐capacity cDNA Reverse Transcriptase Kit, ThermoFisher) according to the manufacturer's instructions. qRT‐PCR was performed using TaqMan Gene Expression Assays (ThermoFisher, see Table S1). The analysis was performed by QuantStudio 3 with the following reaction setup: 50°C for 2 min followed by 95°C for 10 min, 45 cycles at 95°C for 15 s and at 60°C for 1 min. Gene expression data were normalized to endogenous *Gapdh* (Rodent GAPDH Control Reagents, ThermoFisher) and are shown in relation to the naive mice.

### 2.7 Flow cytometry

Standardized liver lobes were mechanically homogenized (chopped by a blade) and incubated in RPMI 1640 supplemented with 10% heat‐inactivated foetal calf serum (FCS), 25 mM HEPES, 100 IU/mL of penicillin, 100 μg/mL streptomycin and 1 mg/mL of collagenase/dispase (Roche, #11097113001) at 37°C for 30 min. The tissue was passed through a 100 μm cell strainer and washed with PBS, and erythrocyte lysis was performed with the RBC lysis buffer (BioLegend). After washing with PBS, the cells were counted by the CASY TT cell counter (OLS‐Omni Life Science).

The cells were stained with Zombie Red Fixable Viability Kit (1:2000; BioLegend) at room temperature for 20 min, and after washing, they were incubated with fluorochrome‐conjugated antibodies (diluted in 3% FCS/PBS) against surface markers at 4°C for 30 min. Two staining panels (“myeloid” and “lymphoid”) were applied, and antibodies and gating strategies are specified in Table S2 and Figures S1 and S2. After washing, the “myeloid” samples were fixed with 2% formaldehyde (4°C, 30 min), permeabilized by 0.05% saponin and incubated with anti‐CD206 antibody (4°C, 30 min). The “lymphoid” samples were fixed and permeabilized by FoxP3 Staining Buffer Set (Miltenyi Biotec, #130‐093‐142) and stained with anti‐forkhead‐box‐protein P3 (Foxp3) antibody (4°C, 30 min). After final washing, the cells were acquired by FACSAria III (BD Biosciences), and the data were analysed by FlowJo (v. 10.0.7).

### 2.8 Hepatic and colonic egg load

Standardized liver lobes or portions of the distal colon were incubated in 4% KOH (37°C, shaking 500 rpm) overnight.^20^ After centrifugation (5 min, 100 *g*), the pellets were resuspended in 400 μL of PBS and *S. mansoni* eggs were counted in 3 × 20 μL aliquots examined under the microscope. The egg load per mg of the tissue was calculated.

### 2.9 Analysis of the parasite phenotype

Mice infected with 300 cercariae were sacrificed 6 wpi, and parasites were collected by perfusion of the portal venous system with RPMI 1640 supplemented by 100 IU/mL of penicillin, 100 μg/mL streptomycin and 1% heparin sodium salt (Merck, #H3149‐100KU). The parasites were separated (males, females and juveniles) under the stereomicroscope and counted, and intact adults were used either in the oviposition test or fixed for further analyses.

To assess the egg‐laying (oviposition) capacity,^21^ males and females isolated from a single mouse (WT FVB/N or *Bsep* ^−/−^) were randomly coupled, placed into 24‐well plates (1 couple/well) and cultivated as described before.^18^ After 3 days, the eggs produced by each couple were counted. To assess the oviposition in different pH, the adults were recovered from NMRI mice (*n* = 3) used for routine life cycle maintenance. Two intact worm pairs per well were cultivated in the physiological blood pH values of *Bsep* ^−/−^ and WT FVB/N mice (6.9 and 7.3, respectively, see Figure 4E); the culture medium pH was adjusted by HCl and NaOH. Egg production was counted after 3‐day cultivation at 37°C in 5% CO_2_.

For morphological analysis, the parasites were fixed in 4% formaldehyde solution, measured, washed with PBS and stained with borax‐carmine overnight. After differentiation in acidic ethanol, they were dehydrated, cleared in methyl salicylate and mounted in Canada balsam. Images of reproductive organs (testes, ovaries) were captured and analysed by ImageJ (v. 1.53). To analyse the surface tegument, the parasites were processed for scanning electron microscopy as described before^22^ and the standardized medial posterior part of parasites was examined.

### 2.10 Statistical analysis

The data were analysed and visualized by GraphPad Prism (v. 9.4). Fisher's exact test, *t*‐test or two‐way ANOVA followed by Šidák's test was used to evaluate differences between the groups as indicated in the figure legends. *p*‐values <.05 were considered significant and are shown as follows: *p < .05, **p < .01, ***p < .001, ****p < .0001 (WT vs. *Bsep* ^−/−^); #*p* < .05, ##*p* < .01, ###*p* < .001 (naive vs. infected). Data are presented as mean ± SD.

## 3 RESULTS

### 3.1 *Bsep* ^−/−^ mice showed milder symptoms after infection with *S. mansoni*

**FIGURE 1:**
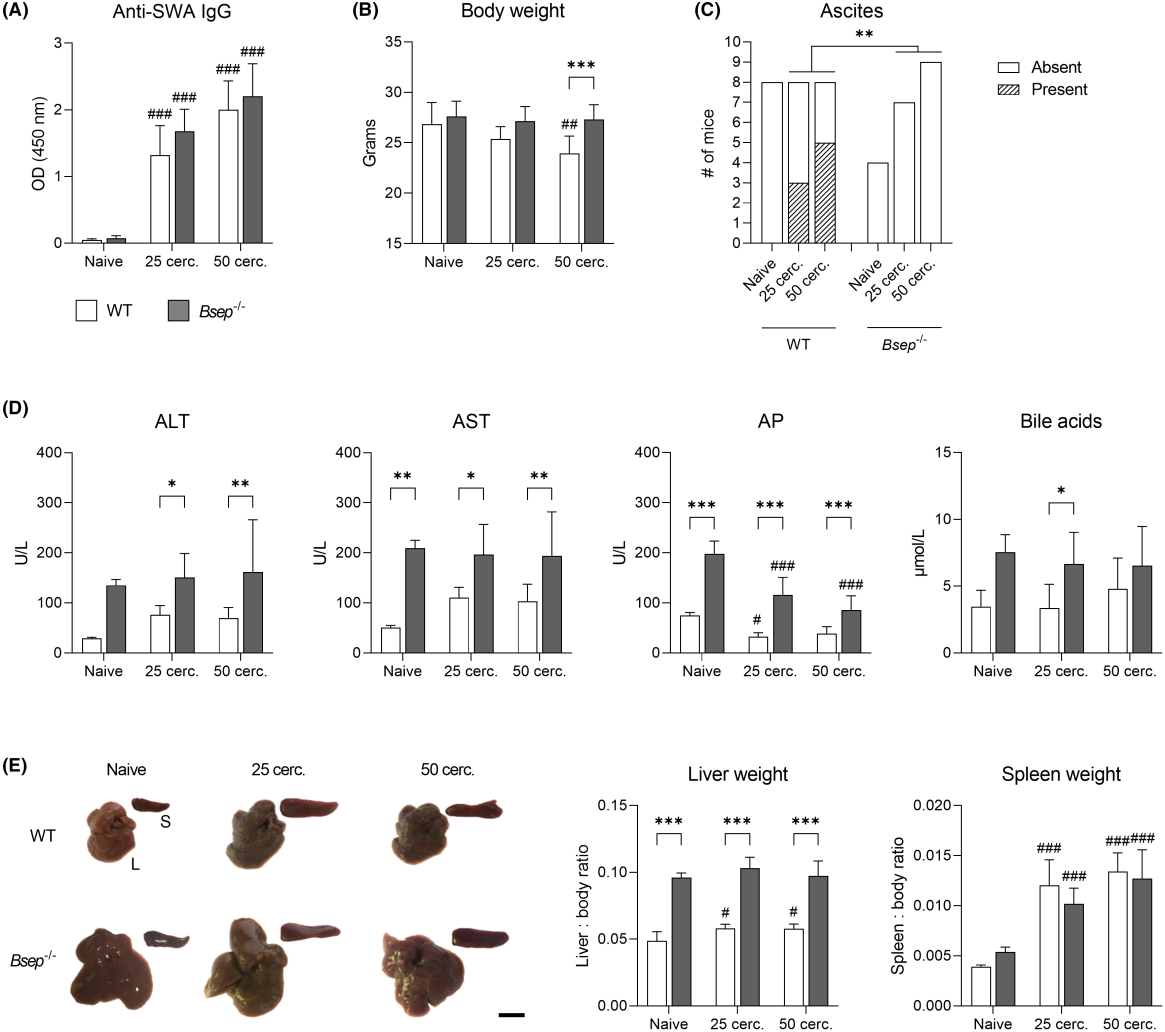
Loss of *Bsep* attenuates symptoms of *Schistosoma mansoni* infection. (A) After 8 weeks of *S. mansoni* infection, both WT and *Bsep* ^−/−^ mice showed similar levels of parasite‐specific serum IgG. (B) While infection with 25 cercariae did not result in weight loss, WT mice infected with 50 cercariae had significantly lower body weight than *Bsep* ^−/−^ mice. (C) In comparison with WT mice, *Bsep* ^−/−^ mice did not develop ascites so frequently. (D) *Bsep* ^−/−^ mice displayed elevated serum levels ALT, AST, AP and BAs compared to WT mice; however, the *S. mansoni* infection did not further increase the aforementioned serum markers. (E) While livers of WT mice enlarged after infection, the organ size remained unaffected in *Bsep* ^−/−^ mice. The spleen size was not affected in *Bsep* ^−/−^ mice compared to WT animals. Scale bar = 1 cm. Quantitative data were evaluated by two‐way ANOVA followed by Šidák's test (A, B, D, E) or Fisher's exact test (C). Statistical significance: *p < .05, **p < .01, ***p < .001 (WT vs. *Bsep* ^−/−^); #*p* < .05, ##*p* < .01, ###*p* < .001 (naive vs. infected).

We first evaluated the general outcome of *S. mansoni* infection in WT and *Bsep* ^−/−^ mice. They both were susceptible to *S. mansoni* infection and mounted comparable levels of parasite‐specific serum IgG (Figure 1A). However, *Bsep* ^−/−^ mice exhibited milder symptoms of the disease. Compared to WT mice, they did not lose weight after infection (Figure 1B) and did not develop signs of ascites, which were detected in half of the infected WT mice (Figure 1C). Corroborating with previous studies,^15^, ^19^, ^23^ *Bsep* ^−/−^ mice had generally elevated serum levels of markers of hepatocellular damage (ALT, AST) and cholestasis (AP, BAs), but this was not worsened after the infection contrary to the tendential increase of ALT and AST in infected WT mice (Figure 1D). In line with these observations, the liver of WT mice was significantly enlarged after infection, but the organ size remained unaffected in *Bsep* ^−/−^ mice (Figure 1E). The spleen size, reflecting the intensity of the systemic host immune response, was not affected in *Bsep* ^−/−^ mice compared with WT animals (Figure 1E).

### 3.2 Hepatic collagen content was reduced in infected *Bsep* ^−/−^ mice

**FIGURE 2:**
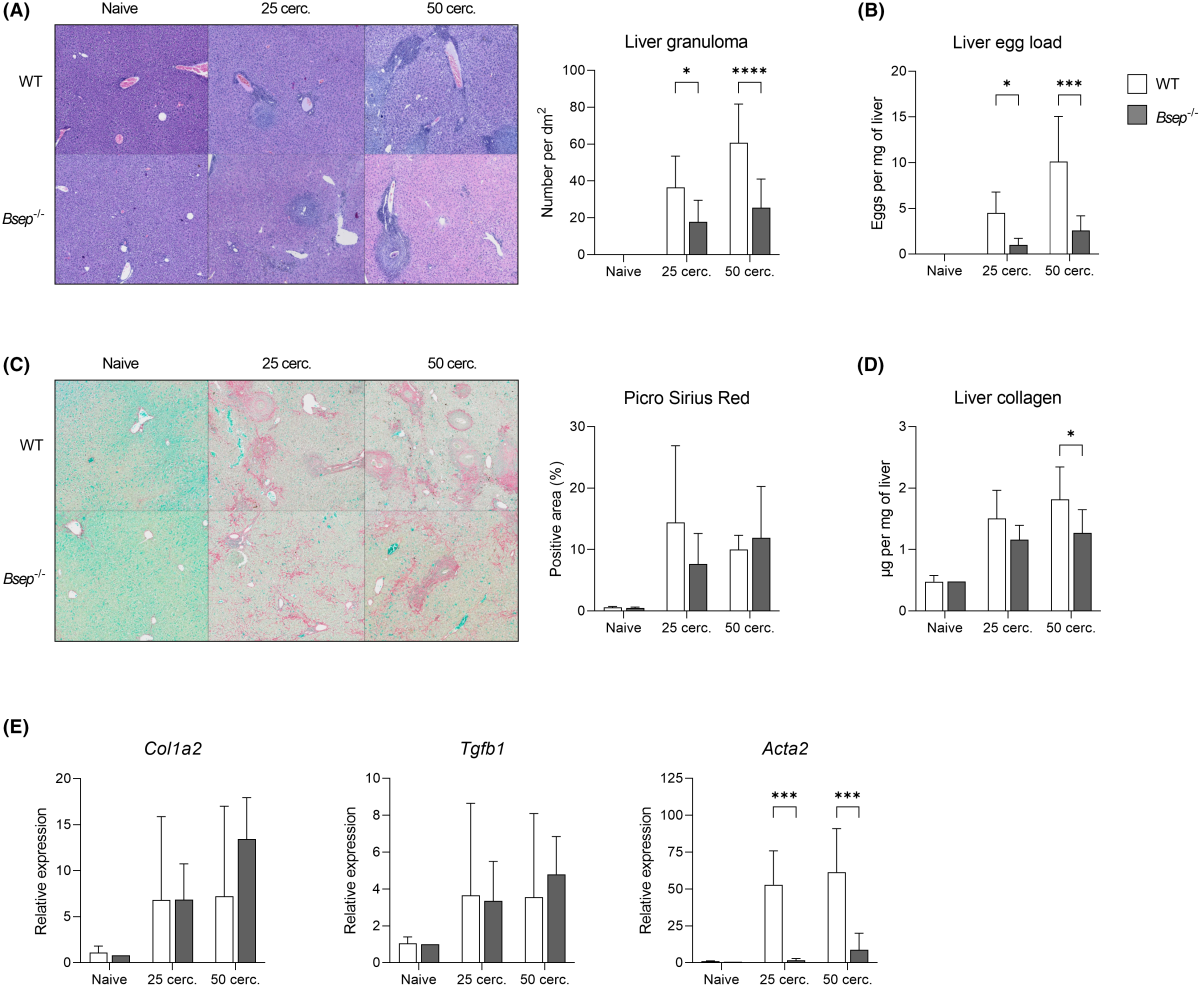
Loss of *Bsep* attenuates *Schistosoma mansoni*‐induced liver fibrosis. (A) H&E staining displays reduced granuloma area in livers of *Bsep* ^−/−^ mice infected either with 25 or 50 cercariae. Enlarged image is available as Figure S5. (B) In livers of *Bsep* ^−/−^ mice, numbers of *S. mansoni* eggs were significantly reduced in mice infected either with 25 or 50 cercariae. (C) Computational analysis of PSR stainings revealed no changes in total collagen area in liver sections. Enlarged image is available as Figure S6. (D) Hydroxyproline assay displayed reduced amount of collagen in liver of *Bsep* ^−/−^ mice infected with 50 cercariae compared to infected WT mice. (E) While gene expression profile of fibrosis markers *Col1a2*, *Tgfb1* remained unaffected in infected *Bsep* ^−/−^ mice, expression of *Acta2*, the marker of collagen‐producing myofibroblasts, was significantly lowered in *Bsep* ^−/−^ mice infected either with 25 or 50 cercariae compared to WT mice. Quantitative data were evaluated by two‐way ANOVA followed by Šidák's test. Statistical significance: *p < .05, ***p < .001, ****p < .0001 (WT vs. *Bsep* ^−/−^).

The formation of granuloma and associated liver fibrosis is the most severe pathological consequence of *S. mansoni* infection. Histological examination revealed the reduction of total granuloma area in infected *Bsep* ^−/−^ mice (Figure 2A). This was caused by the lower amount of *S. mansoni* eggs deposited in the liver (Figure 2B), not the decrease of granuloma size (Figure S3A). Of note, the egg load was diminished also in the colon (Figure S3B), where the eggs are normally excreted, indicating the general reduction of egg production rather than their aberrant dissemination. The computational analysis of the PSR‐positive area did not show differences in total collagen area in liver sections (Figure 2C), but its distribution was partially different in *Bsep* ^−/−^ mice. Specifically, the collagen was found not only in periovular granulomas but also as intercellular collagen dispersed among the hepatocytes (*perisinusoidal fibrosis*), that is not in close proximity to the eggs. While expression of fibrosis markers (collagen 1a2 [*Col1a2* ], *Tgfb1*) remained largely unaffected in *Bsep* ^−/−^ mice (Figure 2E), the hepatic collagen content was decreased (Figure 2D). The latter was in line with the markedly lowered expression of *Acta2* (Figure 2E), the marker of collagen‐producing activated hepatic stellate cells/myofibroblasts.

### 3.3 Infected *Bsep* ^−/−^ mice developed lower degrees of hepatic inflammation

**FIGURE 3:**
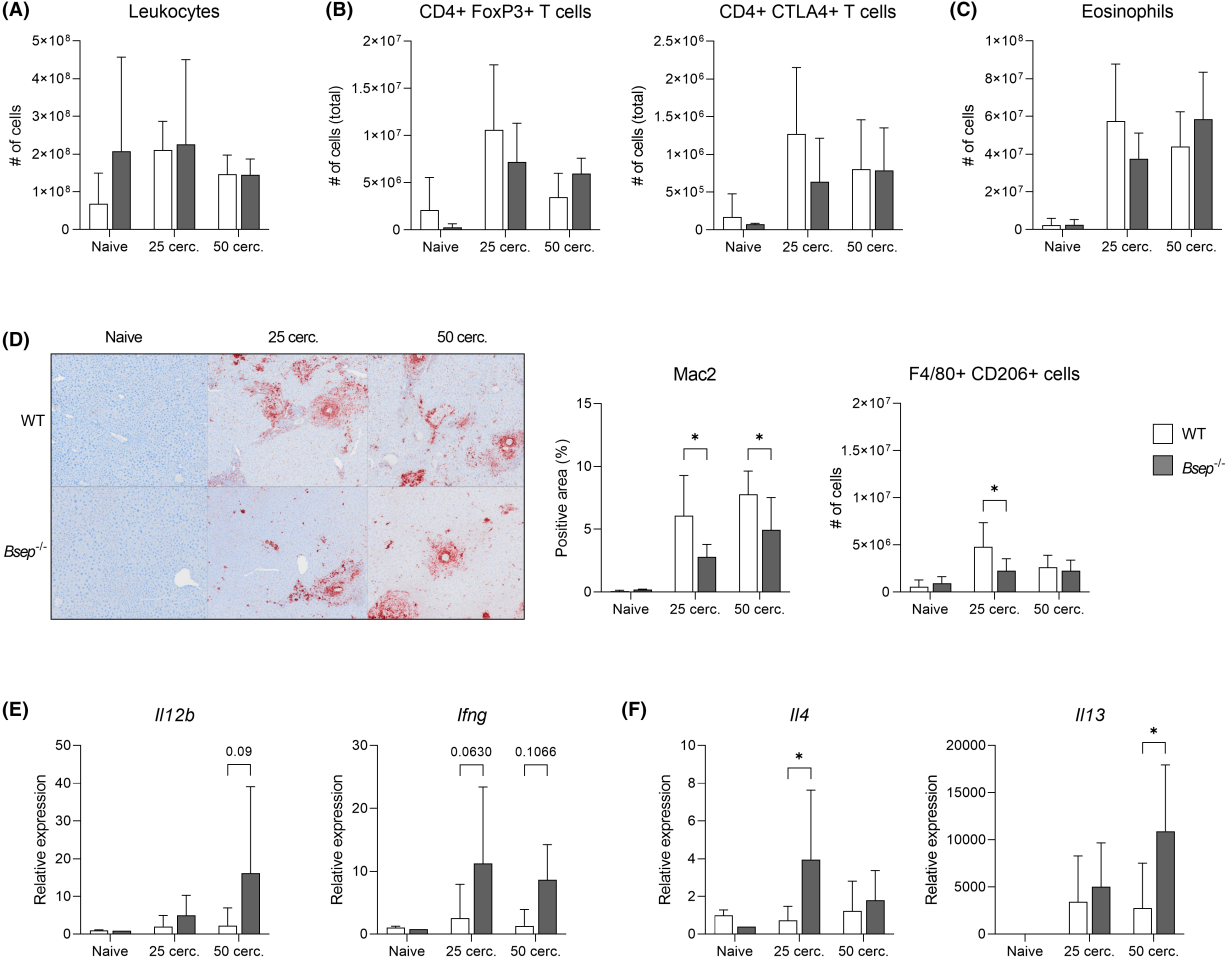
Loss of *Bsep* attenuates *Schistosoma mansoni*‐induced liver inflammation. (A) Flow cytometry analysis revealed similar hepatic leukocyte infiltration in WT and *Bsep* ^−/−^ mice. (B) No significant differences were found for regulatory CD4^+^ Foxp3^+^ T cells or CD4^+^ CTLA4^+^ T cells. (C) Eosinophils were present at similar levels in WT and *Bsep* ^−/−^ mice. (D) Numbers of Mac‐2^+^ cells were reduced in livers of *Bsep* ^−/−^ mice. Enlarged image is available as Figure S7. Accordingly, less F4/80^+^ CD206^+^ cells (M2 macrophages) were found in *Bsep* ^−/−^ mice infected with 25 cercariae. (E) Gene expression of proinflammatory cytokines *Il12b* and *Ifng* was only tendentially increased, while expression of anti‐inflammatory cytokines (*Il4*, *Il13*) (F) was significantly upregulated in infected *Bsep* ^−/−^ mice. Quantitative data were evaluated by two‐way ANOVA followed by Šidák's test. Statistical significance: *p < .05 (WT vs. *Bsep* ^−/−^) or the specific *p*‐value is shown.

Deposition of *S. mansoni* eggs in the liver triggers strong inflammation orchestrated by Th cells. Flow cytometry analysis revealed similar hepatic leukocyte infiltration in WT and *Bsep* ^−/−^ mice (Figure 3A), and no significant differences were observed either for regulatory CD4^+^ Foxp3^+^ T cells or CD4^+^ T cells bearing inhibition receptor cytotoxic T‐lymphocyte‐associated protein 4 (CTLA4; Figure 3B). Eosinophils, prominent innate immune cells infiltrating the liver, were also found in similar levels in WT and *Bsep* ^−/−^ mice (Figure 3C). On the contrary, the Mac‐2^+^ area, indicating the presence of inflammatory leukocytes (especially macrophages) mainly within the periovular granuloma, was reduced in *Bsep* ^−/−^ mice, which also harboured less F4/80^+^ CD206^+^ cells (M2 macrophages) after infection with 25 cercariae (Figure 3D). While the expression of proinflammatory cytokines (*Il12b*, *Ifng*) was only tendentially increased (Figure 3E) or remained unaffected (*Il1b*, *Tnfa*; Figure S4A) in *Bsep* ^−/−^ mice, the expression of anti‐inflammatory cytokines was significantly upregulated (*Il4*, *Il13*; Figure 3F) or tendentially increased (Figure S4B) compared to WT.

### 3.4 *Bsep* ^−/−^ mice impaired development and fecundity of *S. mansoni*

**FIGURE 4:**
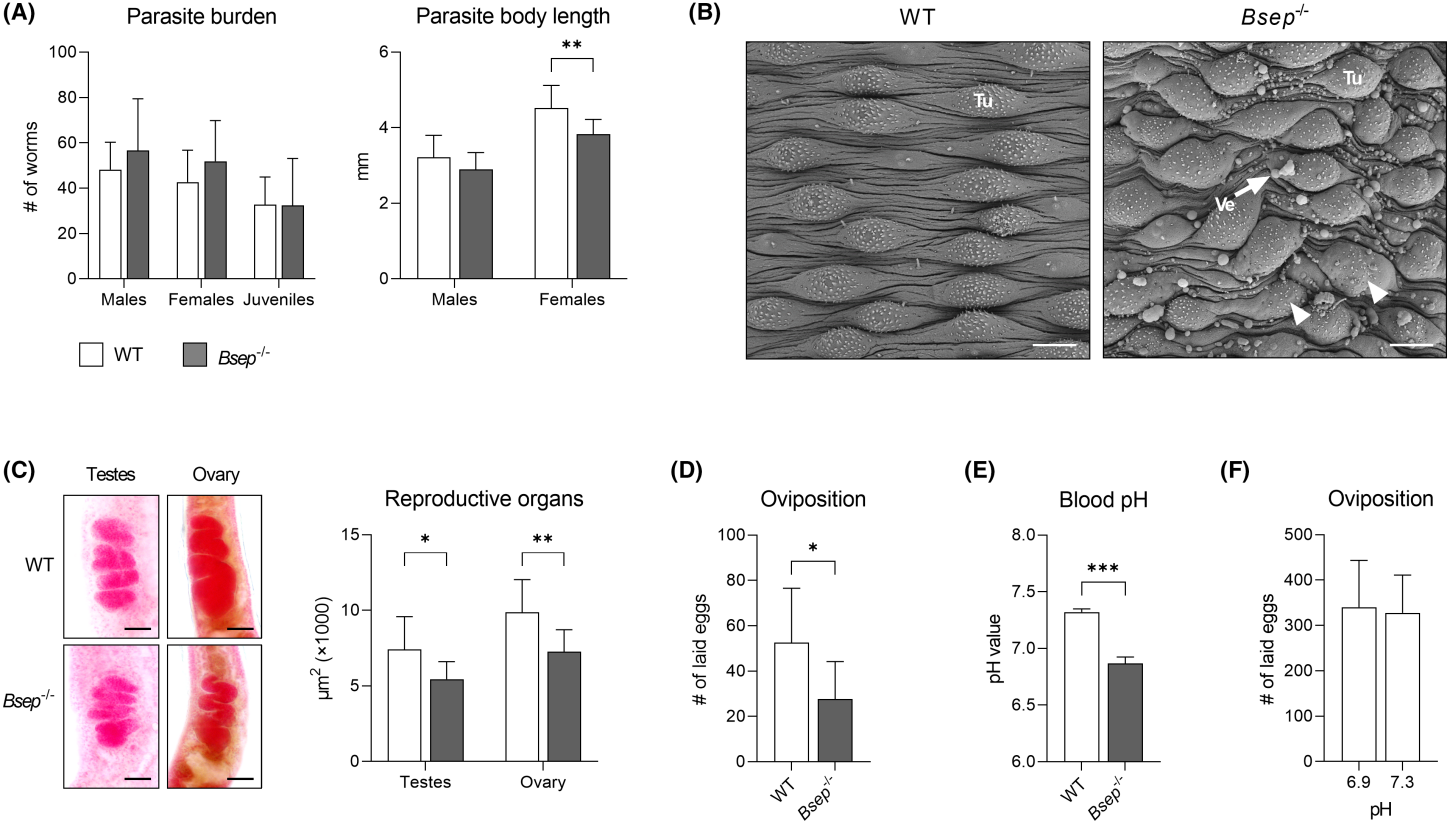
Loss of *Bsep* impairs development and fecundity of *Schistosoma mansoni*. (A) While there was no difference in the number of males, females and juveniles isolated from infected WT and *Bsep* ^−/−^ mice, the adult females isolated from *Bsep* ^−/−^ mice were significantly smaller. (B) Male worms isolated from *Bsep* ^−/−^ mice exhibited alterations of the surface tegument. Vesicles (Ve) budding from the tegument were observed and surface tubercles (Tu) had often less spines, which were disorganized (arrowheads). Scale bar = 10 μm. (C) Microscopic analysis of the parasite reproductive organs showed that the area of the testes and ovaries was reduced in adults isolated from *Bsep* ^−/−^ mice. Scale bar = 50 μm. (D) In vitro, parasites isolated from *Bsep* ^−/−^ mice produced fewer eggs than parasites isolated from WT mice. (E) Blood pH values obtained from *Bsep* ^−/−^ mice were significantly lower compared to WT mice. (F) In vitro production of eggs in pH corresponding to the blood of WT and *Bsep* ^−/−^ mice. Quantitative data were evaluated by *t*‐test. Statistical significance: *p < .05, **p < .01, ***p < .001, (WT vs. *Bsep* ^−/−^).

As our data indicated reduced parasite egg load in *Bsep* ^−/−^ mice, leading to mitigated liver fibrosis and inflammation, we explored the potential mechanisms responsible for this protective effect. Although there were no differences in the number of males, females and juveniles isolated from WT and *Bsep* ^−/−^ mice, the adult females isolated from *Bsep* ^−/−^ mice were significantly smaller (Figure 4A). Moreover, males isolated from *Bsep* ^−/−^ mice exhibited alterations of the surface tegument (Figure 4B). Specifically, vesicles budding from the tegument were observed and the surface tubercles had a lower density of spines, which were less organized than in WT mice. Microscopic analysis of the parasite reproductive organs clearly showed that the total area of the testes and ovaries was reduced in adults isolated from *Bsep* ^−/−^ mice (Figure 4C). To test whether this developmental impairment would have functional consequences on fecundity, the parasites were cultivated in vitro, and egg production was assessed. After 3 days, the parasite isolated from *Bsep* ^−/−^ mice laid significantly fewer eggs (Figure 4D), which was in line with previous in vivo observations (Figures 2B and S3B). Exploring the mechanism responsible for the reduced fecundity, we uncovered that blood pH is generally lower in *Bsep* ^−/−^ mice (Figure 4E). Consequently, we measured the egg production of the parasites isolated from NMRI mice (used for routine life cycle maintenance, not preconditioned with THBAs) in pH 6.9 and 7.3 to simulate *Bsep* ^−/−^ and WT mice blood pH values. Although such a 3‐day in vitro setting did not reveal a significant effect of pH on egg production (Figure 4F), this does not disprove the long‐term effects of lower pH in vivo.

## 4 DISCUSSION

In this study, we investigated the role of THBAs (shown to be protective against cholestatic liver injury^14^, ^15^), the major BA species in *Bsep* ^−/−^ mice,^14^, ^15^ during the development of *S. mansoni*‐induced liver disease.

Our data demonstrate that *Bsep* ^−/−^ mice subjected to *S. mansoni* infection develop no ascites, significant less hepatic inflammation and tendentially less fibrosis in comparison with infected WT mice. Although serum transaminases ALT, AST and AP are markedly elevated in *Bsep* ^−/−^ mice upon *S. mansoni* infection, inflammatory cells such as M2 macrophages (F4/80^+^ CD206^+^ cells) and Mac‐2/galectin‐3^+^ cells (galactose‐specific lectin 3; macrophages, monocytes, dendritic cells, eosinophils, mast cells, natural killer cells and activated T and B cells)^24^, ^25^ were reduced in these animals. Accordingly, mRNA‐expression levels of anti‐inflammatory cytokines such as interleukin (IL)‐4 and IL‐13 were increased in *Bsep* ^−/−^ mice infected with 25 or 50 cercariae, respectively.

While mRNA expression of fibrotic marker *Col1a2* as well as computational analysis of PSR staining remained unchanged among infected WT and *Bsep* ^−/−^ mice, *Acta2* mRNA levels were significantly downregulated in *Bsep* ^−/−^ mice infected either with 25 or 50 cercariae, indicating that despite unchanged formation of collagen fibres due to hepatic egg deposition, the contractility of hepatic stellate cells in infected *Bsep* ^−/−^ mice is reduced.^26^

The mitigated severity of hepatic schistosomiasis in *Bsep* ^−/−^ mice was clearly linked to the reduced hepatic egg load. We demonstrated that impaired parasite fecundity, not the lowered parasite burden, was responsible for the decreased oviposition. Specifically, parasites dwelling in *Bsep* ^−/−^ mice had smaller testes and ovaries, which apparently affected the reproduction rate. Although the host immune attack can block a proper development of schistosome reproductive organs,^27^ we did not observe markedly different immune milieu in *Bsep* ^−/−^ mice. A genome‐wide transcriptome analysis of infected livers, performed at different time points,^4^ might provide deeper understanding of the immune processes in infected WT and *Bsep* ^−/−^ mice.

While the host effector immune response seemed to be unaltered, we detected decreased blood pH and elevated serum concentration of BAs in *Bsep* ^−/−^ mice, which both could impact parasite fecundity, especially from the perspective of long‐term debilitation. As adult schistosomes live directly in the bloodstream, the unfavourable blood pH in *Bsep* ^−/−^ mice can alter the activity of their finely tuned enzymes, such as digestive peptidases processing host blood proteins (e.g. haemoglobin)^28^ or tegumental nucleotidases providing schistosomes with purines.^29^ This would result in impaired nutrient uptake and processing, impeding the proper development of the parasite reproductive system. A similar phenomenon, albeit immune‐mediated, was already shown for other schistosome species invading the nervous tissue.^30^ Moreover, we demonstrated that the tegument of male schistosomes from *Bsep* ^−/−^ mice is morphologically altered suggesting its dysfunction. Being a critical host–parasite interface, facilitating metabolism, signal transduction or immune evasion, the tegument contains water channels, ion transporters or surface receptors.^18^, ^31^ They all can also be affected by the chronically decreased blood pH, leading to aberrant male–female interaction and limited parasite reproduction.^32^

The role of BAs in general (and even more so of THBAs specifically) in schistosome biology is mostly enigmatic. It has previously been shown that the in vitro treatment with UDCA or norUDCA did not harm adult schistosomes^13^ and tauroUDCA treatment even promoted oviposition.^33^ However, these scarce reports originate from short‐term (less than a week) experiments performed in optimal pH and evaluate the effect of single BAs. Although they support the view that BAs may be vital developmental stimuli for a variety of helminths,^34^, ^35^, ^36^, ^37^, ^38^, ^39^, ^40^, ^41^ they can be extrapolated only to a certain extent to our observations. Of note, we noticed rather deleterious effects on schistosomes of the altered serum BAs composition in *Bsep* ^−/−^ mice. This is actually more in line with the studies showing that BAs inhibit glucose uptake^42^ and anaerobic metabolism,^43^ the latter affecting metabolic processes in the parasite's tegument.^44^ The existence of specific BAs transporters, which facilitate helminths survival,^45^, ^46^ also supports the view that some BAs are toxic to helminths and might assist in the host protection. We suggest that a broad transcriptomic comparison of different schistosome life stages (schistosomula, adults and eggs),^47^ isolated from WT and *Bsep* ^−/−^ mice, would shed more light on the effect of altered BA composition on the parasite development and physiology. Moreover, improving the techniques for *S. mansoni* long‐term in vitro cultivation would markedly boost the research of specific factors and stimuli driving or hampering the parasite development.

Despite the lack of direct anthelminthic effects, known from praziquantel (the only approved drug currently available for *S. mansoni* infection in humans),^48^ our finding that altered BA composition and lowered pH values might decelerate the progression of *S. mansoni*‐induced liver injury is of particular clinical interest. In *Bsep* ^−/−^ mice, the protective effect is mediated by decreased parasite fecundity, leading to reduced hepatic egg load, which is the main pathological trigger. Indeed, such a beneficial “anti‐fecundity effect” targeting egg production is also reported and appreciated in the case of schistosomiasis vaccines.^49^ Beyond mitigating tissue pathology, the reduced egg production would result in decreased faecal egg excretion, which is important for disruption of the parasite's life cycle in the environment.^2^ Admittedly, the intentional lowering of blood pH could hardly be translated to clinical practice. Nevertheless, the approach based on the reduction of parasite fecundity could be a promising alternative or support to praziquantel treatment and other drugs possibly mediating such actions should be tested. Since decreased susceptibility to praziquantel of some *S. mansoni* isolates (indicating the emergence of a drug resistance) has already been observed, novel therapeutical strategies are urgently needed.^50^

In conclusion, our study suggests that the altered BA pool and lowered pH values (typical for *Bsep* ^−/−^ mice) may decelerate the progression of *S. mansoni*‐induced liver injury. This could represent a novel approach in the experimental therapy of schistosomiasis.

## FUNDING INFORMATION

MT received funding from the Austrian Science Fund (F7310) and the Medical Scientific Fund of the Mayor of the City of Vienna (MA 40‐GMWF‐485569‐2020). TM was partially funded by the European Funds (ERDF: CZ.02.1.01/0.0/0.0/16_019/0000759, ESF: CZ.02.2.69/0.0/0.0/16_027/0008495).

## CONFLICT OF INTEREST STATEMENT

Michael Trauner served as a consultant for Abbvie, Albireo, BiomX, Boehringer Ingelheim, Falk, Gilead, Genfit, Hightide, Intercept, Jannsen, MSD, Novartis, Phenex, Pliant, Regulus, Siemens and Shire and as a speaker for BMS, Falk, Gilead, Intercept, Madrigal, MSD and Roche. He further received travel grants from Abbvie, Falk, Gilead, Intercept, Jannsen and Roche and unrestricted research grants from Alnlyam, Albireo, Cymabay, Falk, Gilead, Intercept, MSD Takeda and Ultragenyx. He is also co‐inventor of patents on the medical use of *nor*UDCA filed by the Medical University of Graz. Claudia Fuchs received travel grants from Gilead, Roche, Falk, Merck, Vifor, Abbvie and Böhringer Ingelheim. Other authors declare no conflict of interest.

## ETHICS STATEMENT

Animal experiments were performed according to the national (German Society for Laboratory Animal Science) and European (EU Directive 2010/63/EU, Federation of Laboratory Animal Science Associations) guidelines, and they also adhered to ARRIVE guidelines 2.0. The study was approved by the State Office for Agriculture, Food safety and Fisheries of Mecklenburg‐Western Pomerania (7221.3‐1‐011/21).

## Supporting information


Data S1.

## Data Availability

All data generated in the study are presented within the manuscript.
