# Supplementary material for: Bsep/Abcb11 knockout ameliorates Schistosoma mansoni liver pathology by reducing parasite fecundity
Source: Liver Int. 2023 Aug 29;43(11):2469–78. doi: 10.1111/liv.15710 (PMC10947390; doi:10.1111/liv.15710)
Supplement: Supplementary file 1 — Data S1. [file LIV-43-2469-s001.docx]

**Supplementary information to:**

***Bsep*/*Abcb11* knockout ameliorates *Schistosoma mansoni* liver pathology by reducing parasite fecundity**

Tomáš Macháček*^1,2^, Claudia D. Fuchs*^3^, Franziska Winkelmann^1^, Marcus Frank^4,5^, Hubert Scharnagl^6^, Tatjana Stojakovic^7^, Martina Sombetzki^1^, Michael Trauner^3,#^

^1^ Division of Tropical Medicine and Infectious Diseases, Center of Internal Medicine II, Rostock University Medical Center, Rostock, Germany

^2^ Department of Parasitology, Faculty of Science, Charles University, Prague, Czechia

^3^ Hans Popper Laboratory of Molecular Hepatology, Division of Gastroenterology and Hepatology, Department of Internal Medicine III, Medical University of Vienna, Vienna, Austria

^4^ Medical Biology and Electron Microscopy Center, University Medical Center Rostock, Rostock, Germany

^5^ Department Life, Light and Matter, University of Rostock, Rostock, Germany

^6^Clinical Institute of Medical and Chemical Laboratory Diagnostics, Medical University of Graz, Graz, Austria

^7^Clinical Institute of Medical and Chemical Laboratory Diagnostics, University Hospital Graz, Graz, Austria

* These authors contributed equally as joint first authors.

^#^ Corresponding author

**Supplementary Table S1.** TaqMan probes used for the analysis of the gene expression, all purchased from ThermoFisher, Germany.

| Target | Gene name | Assay ID |
| --- | --- | --- |
| *Acta2* | actin, alpha 2, smooth muscle, aorta | Mm00725412_s1 |
| *Col1a2* | collagen, type I, alpha 2 | Mm00483888_m1 |
| *Ifng* | interferon gamma | Mm01168134_m1 |
| *Il1b* | interleukin 1 beta | Mm00434228_m1 |
| *Il4* | interleukin 4 | Mm00445259_m1 |
| *Il10* | interleukin 10 | Mm01288386_m1 |
| *Il12b* | interleukin 12b | Mm01288989_m1 |
| *Il13* | interleukin 13 | Mm00434204_m1 |
| *Tgfb1* | transforming growth factor, beta 1 | Mm01178820_m1 |
| *Tnfa* | tumor necrosis factor | Mm00443258_m1 |

**Supplementary Table S2.** Antibodies used for the flow cytometry analysis.

| Target | Fluorophore | Clone | Vendor | Dilution |
| --- | --- | --- | --- | --- |
| CD3ε | APC | 145-2C11 | BioLegend | 1:120 |
| CD4 | PerCP-Cy5.5 | RM4-4 | BioLegend | 1:200 |
| CD8a | PE-Cy7 | 53-6.7 | BioLegend | 1:600 |
| CD11b | APC | M1/70 | BioLegend | 1:800 |
| CD11c | Alexa Fluor 488 | N418 | BioLegend | 1:400 |
| CD16/CD32 | - | 93 | BioLegend | 1:100 |
| CD19 | Alexa Fluor 488 | 6D5 | BioLegend | 1:150 |
| CD45 | APC-Cy7 | 30-F11 | BioLegend | 1:150 |
| CD206 | PE | C068C2 | BioLegend | 1:80 |
| CTLA4 | PE | UC10-4B9 | BioLegend | 1:100 |
| F4/80 | PE-Cy7 | BM8 | BioLegend | 1:00 |
| FoxP3 | Brilliant Violet 421 | MF-14 | BioLegend | 1:40 |
| SiglecF | PerCP-Cy5.5 | E50-2440 | BD Biosciences | 1:100 |


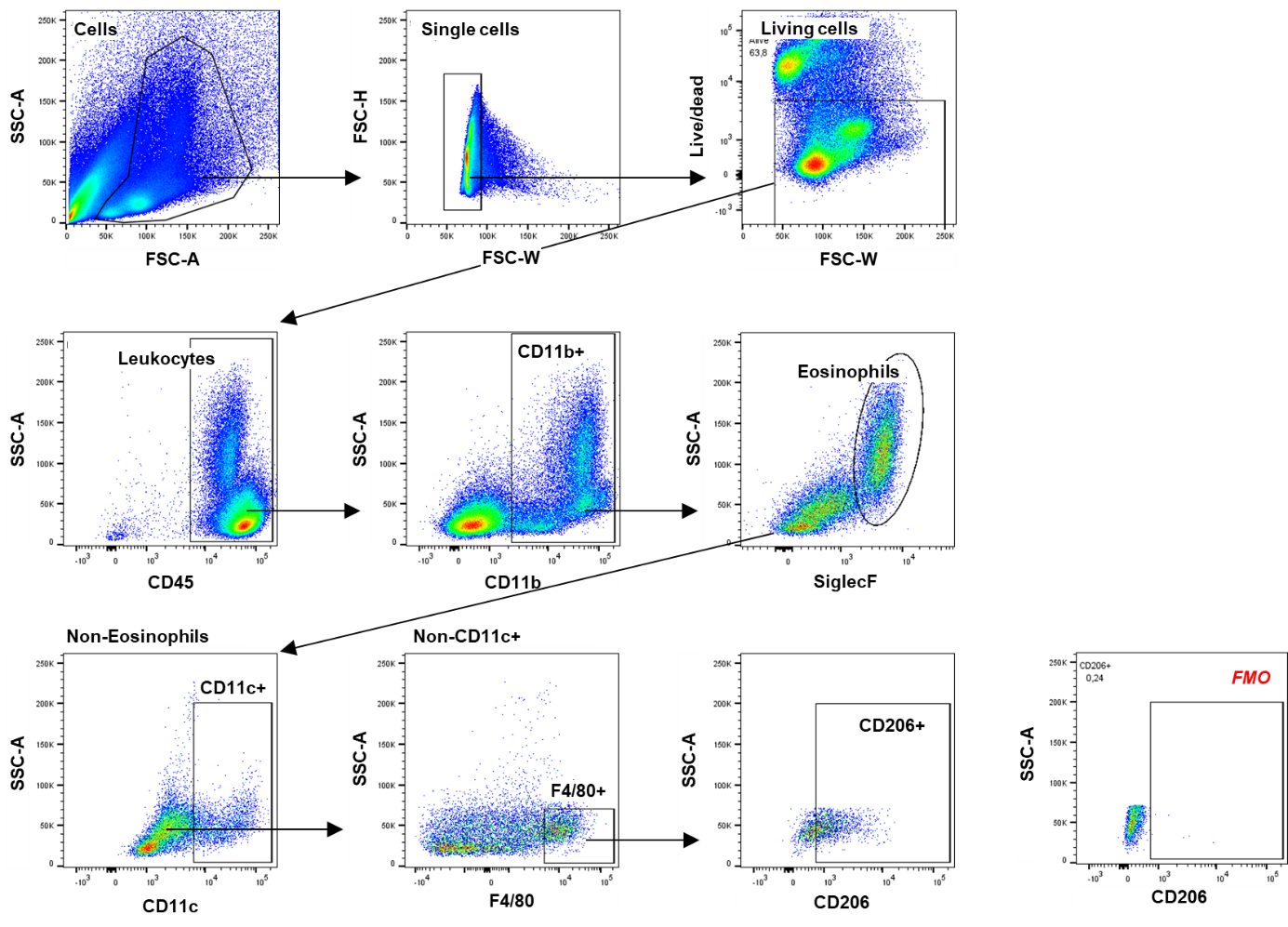


**Supplementary Figure S1.** Gating strategy for myeloid panel.


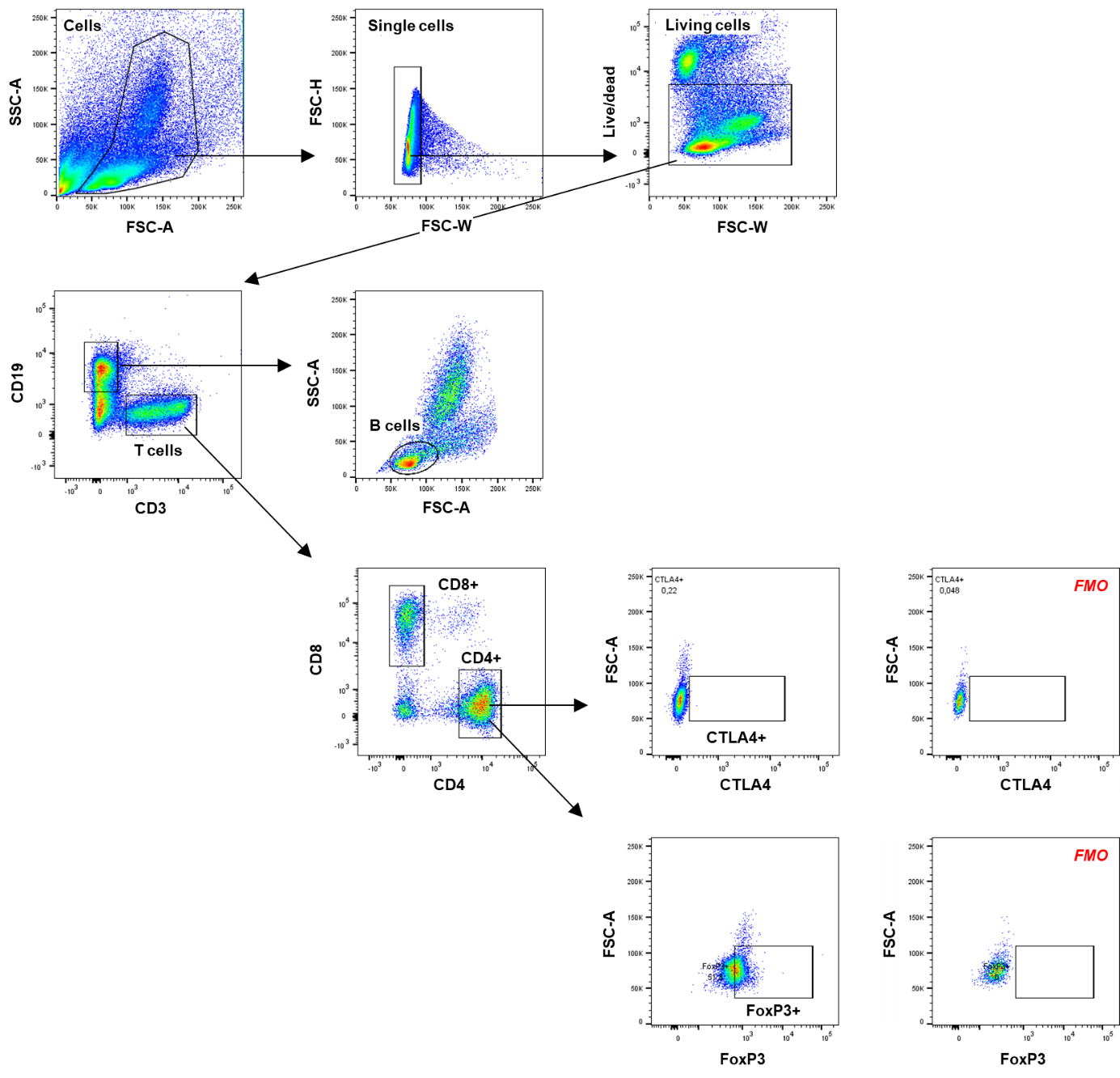


**Supplementary Figure S2.** Gating strategy for lymphoid panel.


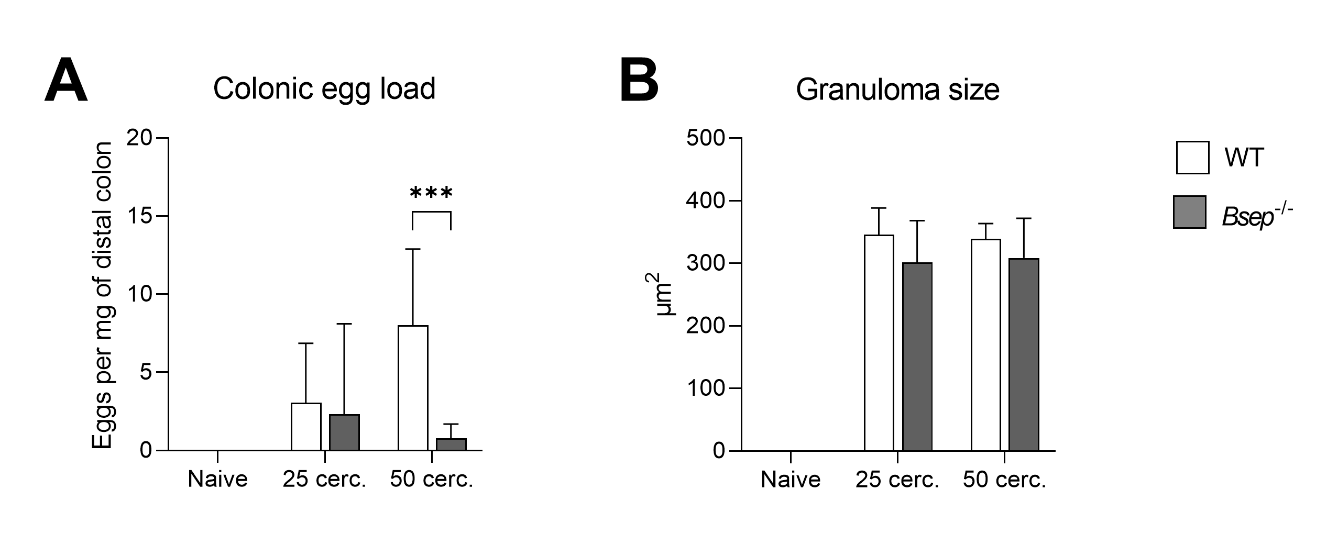


**Supplementary Figure S3.** Colonic egg load **(A)** and size of hepatic granuloma **(B)** in WT and *Bsep*^-/-^ mice 8 weeks post infection with *S. mansoni*. Statistical significance: ***p<0.001 (WT vs. *Bsep*^-/-^).


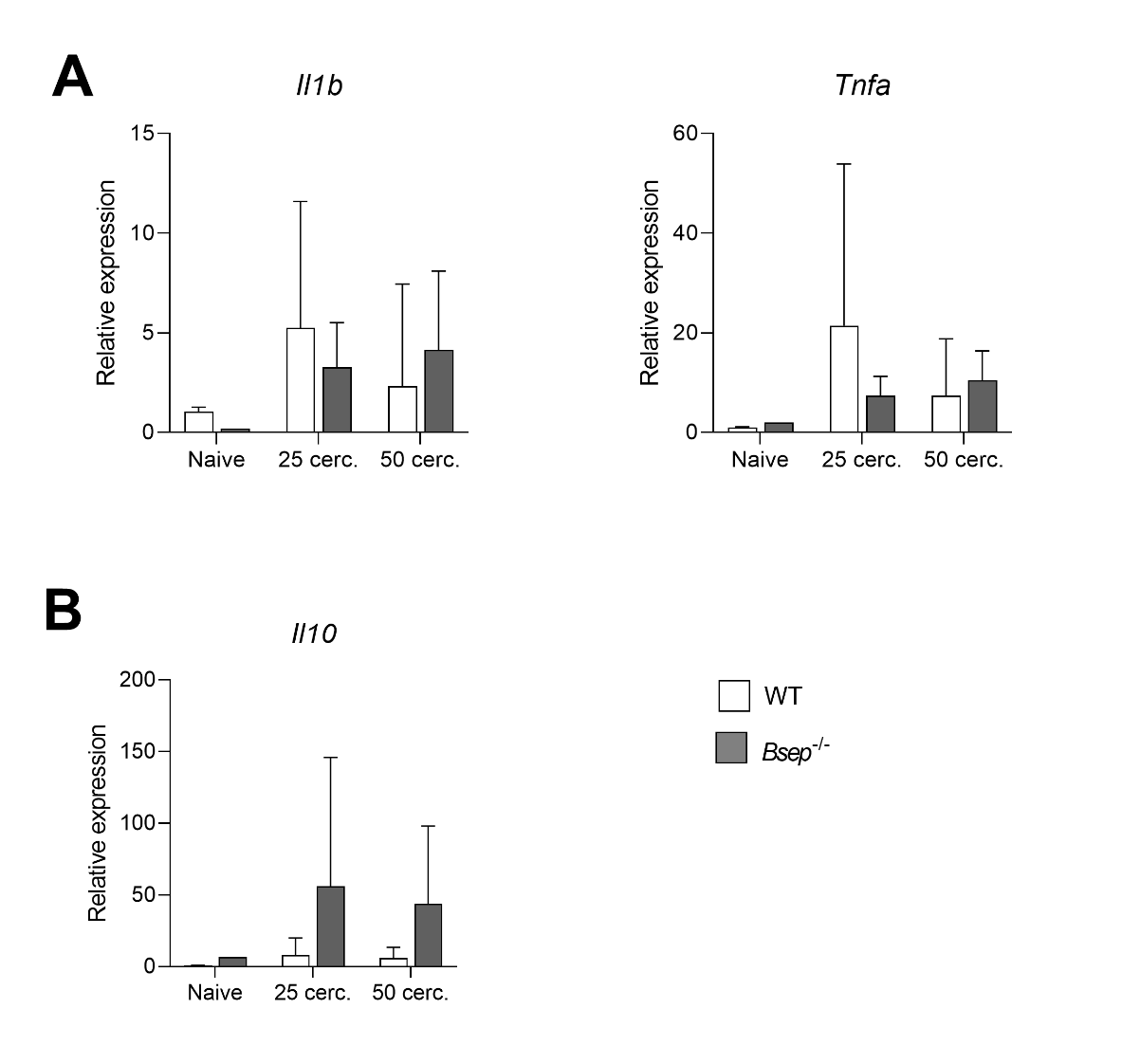


**Supplementary Figure S4.** Expression of inflammatory **(A)** and regulatory **(B**) cytokines in the liver of WT and *Bsep*^-/-^ mice 8 weeks post infection with *S. mansoni*.


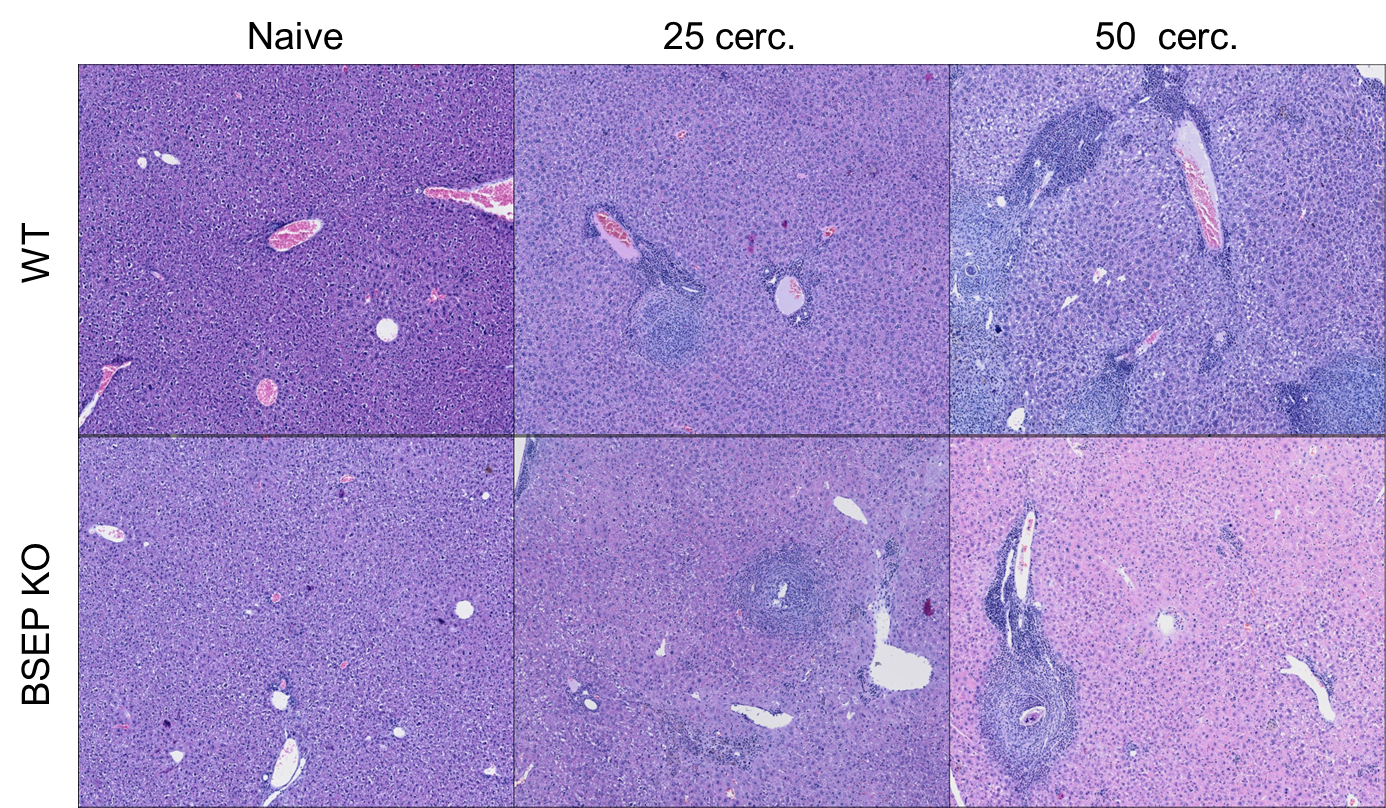


*Bsep*^-/-^

**Supplementary Figure S5.** Enlarged version of the image presented in Fig. 2A. H&E staining displays reduced granuloma area in livers of *Bsep*^-/-^ mice infected either with 25 or 50 cercariae.


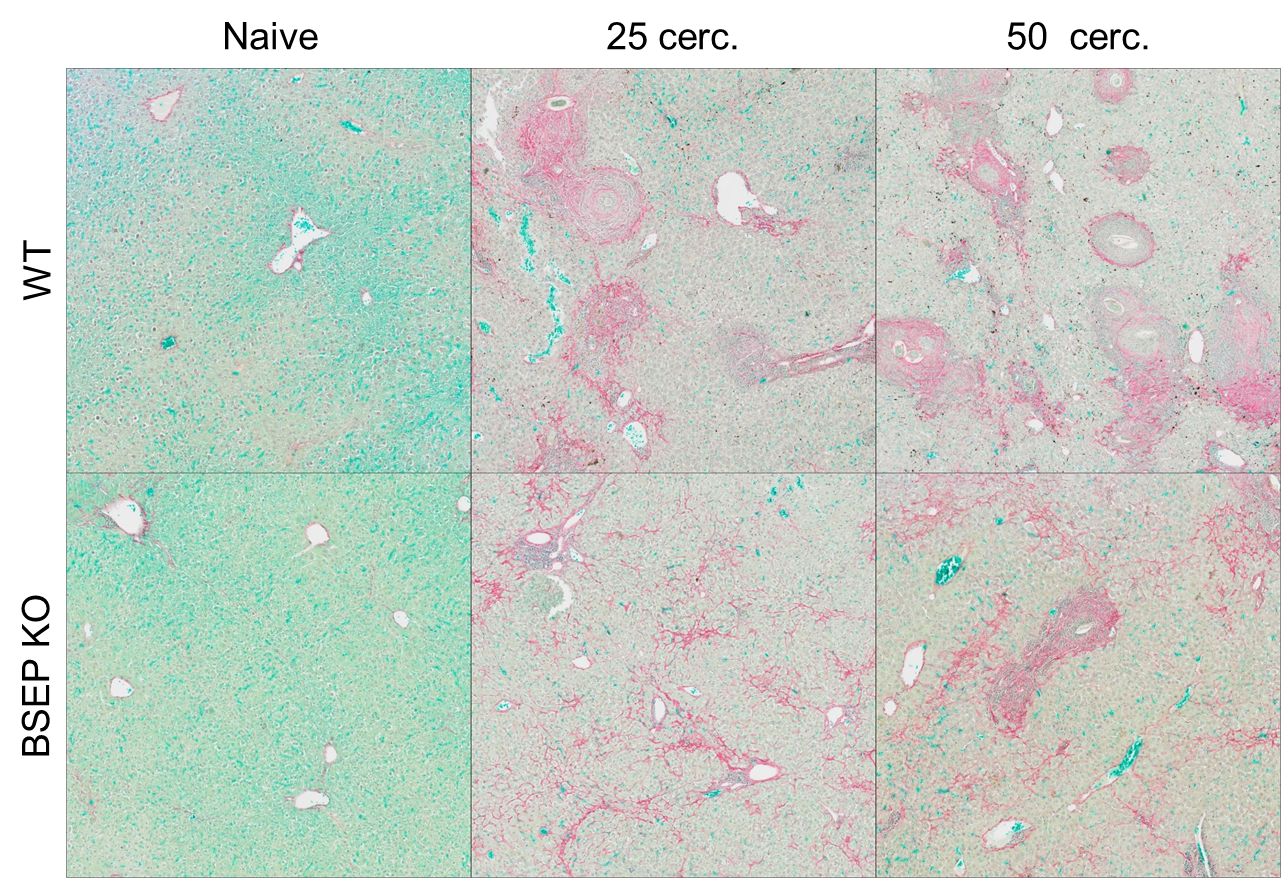


*Bsep*^-/-^

**Supplementary Figure S6.** Enlarged version of the image presented in Fig. 2C. Computational analysis of Picro Sirius Red staining revealed no changes in total collagen area in liver sections.


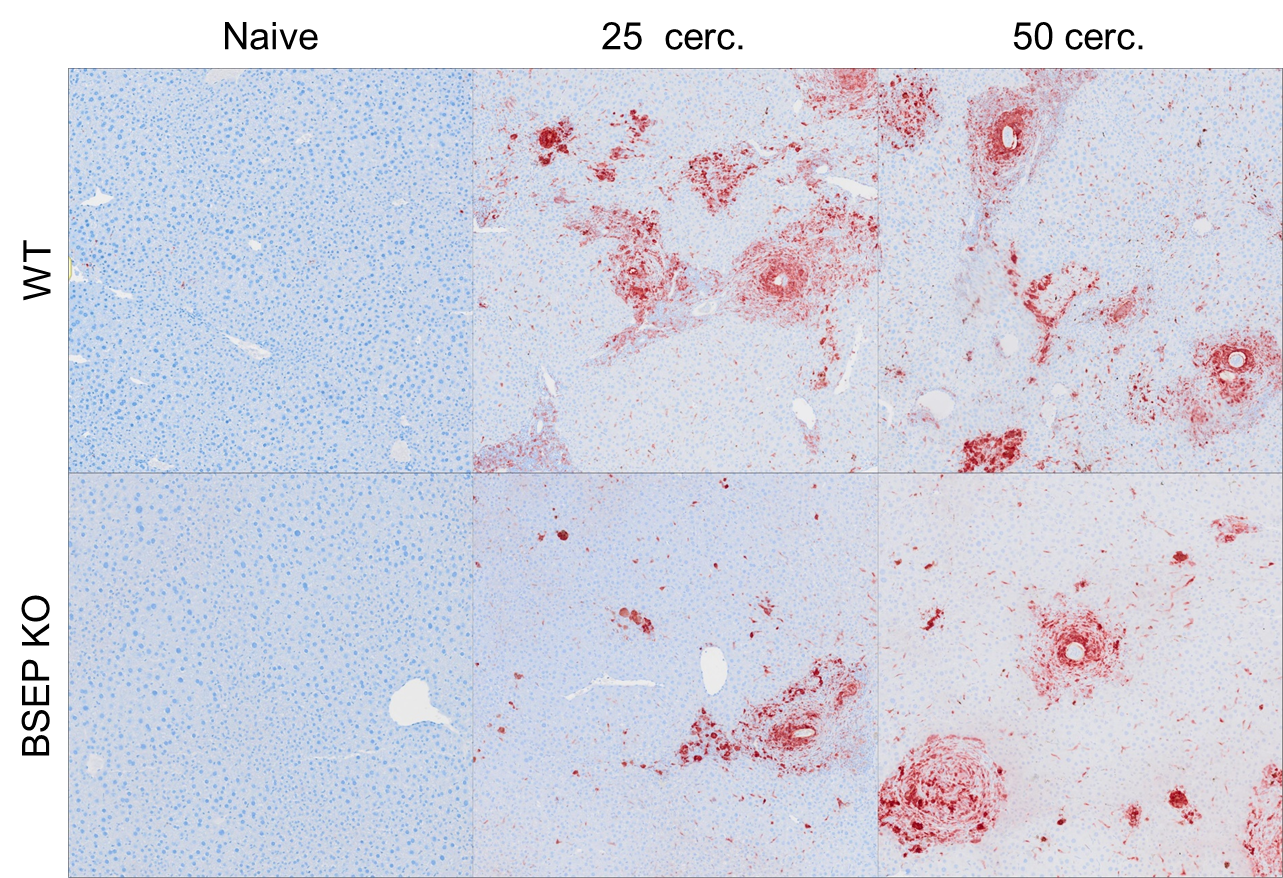


*Bsep*^-/-^

**Supplementary Figure S7.** Enlarged version of the image presented in Fig. 3D. Numbers of Mac-2+ cells were reduced in livers of *Bsep*^-/-^ mice.
